# Investigating changes in student mental health and help-seeking behaviour after the introduction of new well-being support services at a UK university

**DOI:** 10.1192/bjo.2024.711

**Published:** 2024-05-27

**Authors:** Jacks Bennett, Claire M. A. Haworth, Judi Kidger, Jon Heron, Myles-Jay Linton, David Gunnell

**Affiliations:** Population Heath Sciences, Bristol Medical School, University of Bristol, UK; School of Psychological Science, University of Bristol, UK

**Keywords:** University, mental health, well-being, students, support services

## Abstract

**Background:**

Growing numbers of students now seek mental health support from their higher education providers. In response, a number of universities have invested in non-clinical well-being services, but there have been few evaluations of these. This research addresses a critical gap in the existing literature.

**Aims:**

This study examined the impact of introducing non-clinical well-being advisers on student mental health and help-seeking behaviour at a large UK university.

**Method:**

Survey data collected pre–post service introduction in 2018 (*n* = 5562) and 2019 (*n* = 2637) measured prevalence of depression (Patient Health Questionnaire-9), anxiety (Generalised Anxiety Disorder-7), and low mental well-being (Warwick–Edinburgh Mental Wellbeing Scale), alongside student support-seeking behaviour. Logistic regression models investigated changes in outcome measures. Administrative data (2014–2020) were used to investigate corresponding trends in antidepressant prescribing at the onsite health service, student counselling referrals and course withdrawal rates.

**Results:**

Adjusted models suggested reductions in students’ levels of anxiety (odds ratio 0.86, 95% CI 0.77–0.96) and low well-being (odds ratio 0.84, 95% CI 0.75–0.94) in 2019, but not depression symptoms (odds ratio 1.05, 95% CI 0.93–1.17). Statistical evidence showed reduced student counselling referrals, with antidepressant prescribing and course withdrawal rates levelling off. Student perception of the availability and accessibility of university support improved.

**Conclusions:**

Our findings suggest a non-clinical well-being service model may improve student perception of support, influence overall levels of anxiety and low well-being, and reduce clinical need. The current study was only able to examine changes over the short term, and a longer follow-up is needed.

## Student mental health, well-being and support-seeking

The number of university students seeking mental health support during their studies has increased significantly both in the UK and internationally over the past decade.^1,2^ Students face unique academic, financial and social challenges, with global research suggesting more than a third will have experienced a common mental health disorder at some point in their lives.^3–5^ Obtaining reliable estimates of prevalence is challenging: prevalence is influenced not only by environmental and individual factors, but also greater mental health awareness and disclosure, meaning more students may be willing to seek help.^6,7^

## Mental health and well-being support services in higher education

With increased demand for mental health and well-being support, UK higher education providers have broadened their welfare service provision to include student health and counselling services, disability services, accommodation teams, peer mentors, non-clinical well-being advisers and online support.^8,9^ Despite extensive evaluation of individual psychological, psychoeducation or online student support interventions, the use of differing study designs, outcome measures and heterogenous samples has resulted in a mixed and often conflicting evidence base.^10–12^ Although there is some evidence for the effectiveness of student counselling services, particularly for treating depression, anxiety and low well-being, there is little research examining the impact of non-clinical well-being advisory support, accommodation well-being teams or settings-based and organisational-level strategies.^13–15^ Higher education providers are under increasing pressure to offer evidenced-based, ‘whole-university’ mental health support, i.e. taking institutional, holistic and preventative approaches, recently advocated by Universities UK and the Student Mental Health Charter.^16,17^ Consequently, examination of broader student well-being support models is critically needed.^18,19^

## Evaluating student mental health and well-being support

Evaluation of institution-level health interventions in education is challenging. Gold-standard randomised controlled trials are expensive and often unfeasible, with rare high-quality examples in schools indicating need for considerable resources.^20^ Current Medical Research Council guidance supports the adoption of flexible methodology.^21^ The current study took advantage of a natural experiment at one UK university in 2018, following a step-change in its student mental health and well-being provision. It offered an opportunity to examine the impact of introducing new well-being support at a single point in time, in a large student population.^22^

## Local context and aims

**Fig. 1:**
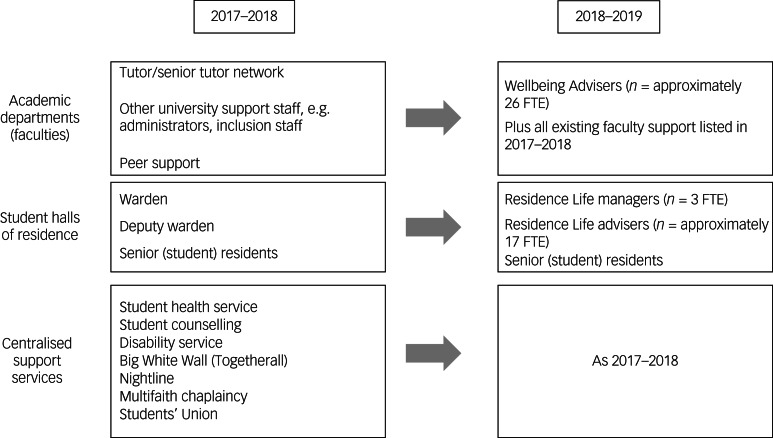
Institution's key student pastoral and support services. *Previous and general staffing totals were not available. FTE, full-time equivalent.

Before the new investment, this particular institution had become a media focus for national concerns about growing levels of student mental health difficulties and wait times for student counselling services.^23^ In line with a ‘whole-university’ framework, it increased its annual mental health budget by an additional £1 million in the academic year 2018–2019, creating more than 40 non-clinical well-being adviser roles across academic departments and in university student accommodation (in 2017–2018, the institution was made up of 25 academic schools organised into six broader disciplines known as faculties: arts, engineering, health sciences, life sciences, science, social science and law).^16,24^ Figure 1 shows that previous welfare support in academic departments had primarily relied on pastoral academic tutors/supervisors and administrators, alongside centralised support, e.g. designated student counselling services and an onsite general practice. The addition of new well-being advisers in academic departments (around 26 new staff roles) was designed to relieve pressure on academic staff and existing clinical/professional services, but not replace them. The new well-being model in student accommodation was a restructure: it saw designated, professional well-being advisers (‘Residence Life’; an anonymised generic name for the well-being advisers in student accommodation) operating 24 h a day out of three central campus hubs. The team (approximately 20 newly recruited staff) replaced a previous ‘warden system’, in which academics had provided live-in welfare support in individual halls of residence (academic wardens in (approximately 30) halls of residences provided oversight and pastoral care alongside their teaching and research commitments, supported by a team of senior student residents) (see Fig. 1).

Rather than grow existing support services, the ambition in both academic departments and student accommodation was to add stepped-care, low-intensity, professional well-being support that would address student issues early before they developed into mental health difficulties, signpost students to appropriate resources and/or liaise with academics and other services, improve pathways into student counselling or other resource-intensive support for those with more severe concerns, and deliver proactive well-being initiatives.^24^ Staff recruited to the new well-being roles were not required to have a clinical qualification, and received internally and externally delivered mental health training (see Supplementary File 1 available at https://doi.org/10.1192/bjo.2024.711). During the first year of operation, the Residence Life and Well-being services provided individual support to over 5000 students (i.e. more than one in six students).^24^

The overall aim of this study was to examine changes in student population mental health, well-being and help-seeking behaviour before and after the introduction of new university well-being services. We examined the following outcomes: (a) overall levels of student depression, anxiety and low mental well-being; (b) mental health differences by sociodemographic, health and education factors; (c) student use of university support; (d) perceived barriers to seeking university support; (e) numbers of antidepressants prescribed at the onsite student health service; (f) student counselling service referrals; and (g) student course withdrawals for mental health reasons.

## Method

### Design

The current study contributed to a broader mixed-methods evaluation of the new well-being services, which included staff and student focus groups and interviews, alongside well-being service use data.^25^ The research took place in a university in South-West England, where, in 2018–2019, there were approximately 27 000 students and 6000 staff. A total of 20% of registered students were international; 27% were from Black, Asian and minority ethnicity backgrounds; and 74% were undergraduates. More than 8000 (mostly first year) students lived in university halls of residence across the city.

Changes in mental health and help-seeking behaviour were investigated with cross-sectional student survey data collected in the summer terms of 2017–2018 and 2018–2019, i.e. pre–post new well-being service introduction. With no comparator universities, the original aim was to examine a longer time-series of post-intervention years; however, the COVID-19 pandemic (onset in March 2020) led to radical changes in service delivery and context, therefore only survey data to 2019 were considered. Nevertheless, routinely collected administrative student data were used to place the survey findings in a wider context of student mental health experience, spanning 2014 to 2020.

### Ethical approval

The authors assert that all procedures contributing to this work comply with the ethical standards of the relevant national and institutional committees on human experimentation and with the Helsinki Declaration of 1975, as revised in 2008. All procedures were approved by the Institution's Health Sciences Ethics Committee (reference 85483). Participant information at the beginning of the survey outlined the aims and risks of the study, with relevant support contacts offered throughout. All respondents gave written (electronic) informed consent to taking part in research.

### Procedure and measures

#### Survey data

All registered undergraduates and postgraduates at the institution were invited by email and social media to take part in anonymous, 15-min, online student well-being surveys in the summer terms of 2018 and 2019. The 2018 survey was open from 30 April to 21 June (*n* = 24 915 registered students); the 2019 survey was open from 6 to 27 May (*n* = 26 053). Supplementary File 2 details the survey measures used in the current study and rationale for their use, including validated screens of depression symptoms (Patient Health Questionnaire-9 (PHQ-9)),^26^ anxiety symptoms (Generalised Anxiety Disorder-7 (GAD-7))^27^ and mental well-being (the 14-item Warwick–Edinburgh Mental Wellbeing Scale (WEMWBS)).^28^ Support experience questions included which sources of university support students had used (see Supplementary File 2) and any perceived structural/perceptual barriers to seeking help (items adapted from previous research).^29^ Sources of support response options included staff in residences, well-being advisers, personal tutors/academic mentors, general practitioners/doctors, mental health professionals (including psychiatrist or psychologist, counsellor or social worker), peer support, other university staff, online/telephone services and the Students’ Union (see Supplementary File 2*).* Barriers to help-seeking included lack of confidentiality, concern no one would understand, did not know where to find help, fear of unwanted intervention, stigma, lack of available services and difficulty with access (Supplementary File 2). Other survey items included sociodemographic characteristics, educational factors and health factors, including assessment of previous or current mental health difficulties (i.e. lifetime mental health diagnosis) (see Supplementary File 2).

#### Administrative data

We examined change in numbers of selective serotonin reuptake inhibitors (SSRIs) antidepressants prescribed at the onsite student health service between September 2014 and February 2020 (no data from after March 2020 was used because of COVID-19-related disruption and UK campus closures), on the basis that the new well-being support might mitigate need for students to seek medical help. SSRIs are often the first-line treatment for symptoms of depression or anxiety.^30^ The local clinical commissioning group provided monthly prescription totals for citalopram, escitalopram, fluoxetine, paroxetine and sertraline. The student health service provided numbers of practice registered students for each academic year, from September to August (2014–2020).

We also investigated demand for the student counselling service between academic years 2014–2015 and 2018–2019, since organisational rationale for investment in non-clinical adviser support had been to help reduce high-intensity mental health referrals.^24^ Data were provided by the student counselling service as unique referral/registration totals between October and September each academic year.

We examined numbers of students withdrawing from their courses between 2014–2015 and 2018–2019 on the basis that new well-being support might have prevented more students leaving for mental health reasons. Institutional data provided annual course withdrawal totals specifically citing mental health reasons.

### Analyses

#### Survey data

Descriptive statistics (frequencies and percentages) were used to describe and compare survey samples with institution data to assess representativeness of respondents. Stata version 16 for Windows was used for all analyses.^31^

#### Depression, anxiety and well-being

Depression, anxiety and mental well-being scores are reported as dichotomous outcomes, i.e. percentage scoring above/below recommended clinical cut-offs (see Supplementary File 2). Continuous outcomes (means and s.d.) are reported to enable future cross-sector comparison, but not analysed further here. Multivariable logistic regression models examined change in odds of moderate/severe student depression and anxiety symptoms (i.e. scoring >10) and low mental well-being (i.e. scoring <42) across the 2018 and 2019 survey years. To account for differing response rates and sample characteristics in each survey, we used adjusted models to control for 12 sociodemographic-, health- and education-related factors, also previously identified for their associations with student mental health outcomes (Supplementary File 2). The unadjusted estimates are presented for comparison. Survey item response missingness was generally low (0.0–4.1%) – an exception was whether a student had experienced a lifetime mental health diagnosis in 2018 (16.7%) compared with 2019 (0.5%), which was caused by the questionnaire format. Sensitivity analyses suggested the missingness was non-systematic (see Supplementary File 3), therefore lifetime mental health diagnosis was included in the adjusted models.

#### Mental health outcomes stratified by students’ sociodemographic, health and educational characteristics

Multivariable logistic regression models were used to examine any changes which differed across student sociodemographic groups, i.e. to see whether any improvement or deterioration between 2018 and 2019 was restricted to particular student groups. Interaction analyses included all previous confounders, except age and residence, because of collinearity with year of study.

#### Help-seeking behaviour

Frequencies and percentages are used to describe student use of different sources of university support in 2018 and 2019 (see Supplementary File 2). It was not possible to directly compare student use of the new support, as well-being advisers in academic departments were only available from the academic year 2018–2019. Likewise, reporting of support use in student accommodation is limited to help-seeking from staff in residences, rather than explicit comparison of wardens and Residence Life staff. Reporting was restricted to students in their first year of study (including undergraduates and postgraduates) (*n* = 1817 in 2018; *n* = 952 in 2019), to avoid individual students reflecting on more than one year.

#### Barriers to university support

The number and nature of reasons that students gave for not seeking university support (i.e. perceived barriers) are reported as frequencies and percentages. These analyses included all responding students (*n* = 8199), students in their first year of study (*n* = 2769) and students showing severe major depression (*n* = 695; i.e. the most vulnerable, with PHQ-9 scores of >20).^26^ Respondents could tick all barrier options that applied (Supplementary File 2); those reporting ‘no problem*’* were omitted from further analyses. Logistic regression models were then used to examine change in perceived barriers to seeking university support for the three groups between 2018 and 2019.

#### Administrative data

##### Antidepressant prescribing

We compared 6-monthly SSRI antidepressant prescription totals per 1000 registered students between September 2014 and February 2020, presenting findings graphically. We also descriptively report annual percentage change in SSRI items prescribed.

##### Student counselling referrals

We examined numbers of unique student counselling service referrals per 100 university-registered students between 2014–2015 and 2018–2019, plotting totals graphically. We calculated risk ratios by using a Stata *iri* command (https://www.stata.com/features/overview/tables-for-epidemiologists), to estimate change in referral rates between 2017–2018 and 2018–2019, i.e. pre–post well-being service introduction.

##### Student course withdrawals

We examined annual withdrawal totals citing mental health reasons (per 1000 registered students) for students in their first year of study and all students (including first year), reporting totals graphically. Risk ratios for mental health course withdrawals between 2017–2018 and 2018–2019 were also compared for both groups.

## Results

### Survey findings

#### Sample characteristics

Survey sample sizes and response rates differed between 2018 (*n* = 5562/24 915; 22.3%) and 2019 (*n* = 2637/26 053; 10.1%). Table 1 shows sample characteristics, distribution of missing data and comparison with institution data. The samples were generally well-matched and broadly representative of the eligible student population, with some exceptions. Survey respondents identifying as female, minority gender, White ethnicity or reporting a disability were overrepresented compared with the whole student population; similarly, there were more undergraduate and home students (although this was less marked in 2019). The proportion of respondents aged ≥21 years was greater than seen in institution data, and fewer were in their first year of study compared with registry data.

**Table 1:** Sample characteristics compared to institution data (totals do not equal 100% because of rounding)

Year	2018	2019	Institution data 2017–2018	Institution data 2018–2019
Number of respondents/eligible students	5562	2637	24 915	26 053
Response rate (%)	22.3	10.1		
Gender, *n* (%)				
Female	3614 (65.0)	1829 (69.4)	13 755 (55.2)	14 520 (55.7)
Male	1829 (32.9)	720 (27.3)	11 107 (44.6)	11 476 (44.0)
Nonbinary or another gender	62 (1.1)	28 (1.1)	52 (0.2)	53 (0.2)
Prefer not to say/missing	57 (1.0)	60 (2.3)	0.0	0.0
Age, *n* (%)				
<21 years	2658 (47.8)	1122 (42.6)	17 322 (69.5)	18 060 (69.3)
≥21 years	2677 (48.1)	1486 (56.4)	7595 (30.5)	7997 (30.7)
Missing	227 (4.1)	29 (1.0)	Not applicable	Not applicable
Ethnicity, *n* (%)				
Black, Asian, minority ethnicity	952 (17.1)	528 (20.0)	6339 (25.4)	7087 (27.2)
White ethnicity	4503 (80.1)	2072 (78.6)	16 992 (68.2)	17 239 (66.2)
Non-disclosed/missing	107 (1.9)	37 (1.4)	1584 (6.4)	1727 (6.6)
Sexual orientation, *n* (%)				
Heterosexual	4364 (78.5)	1968 (74.6)	Not applicable	Not applicable
Lesbian, gay, bisexual or prefer to self-describe (LGBTQ)	958 (17.2)	492 (18.7)		
Prefer not to say/missing	240 (4.3)	177 (6.7)		
Fee status, *n* (%)^a^				
Home	4847 (87.9)	2129 (80.7)	20 254 (81.3)	20 816 (79.9)
European Union	273 (4.9)	196 (7.4)		
International students	393 (7.1)	307 (11.6)	4654 (18.7)	5233 (20.1)
Missing	49 (0.9)	5 (0.2)	7 (0.0)	4 (0.0)
Course level, *n* (%)				
Postgraduate research/taught	655 (11.8)	593 (22.5)	6498 (26.1)	6910 (26.6)
Undergraduate	4867 (87.5)	2041 (77.4)	18 423 (73.9)	19 151 (73.5)
Missing	40 (0.7)	3 (0.1)	Not applicable	Not applicable
Previous education, *n* (%)				
State/grammar (non-fee)	3346 (60.1)	1685 (63.8)	Not applicable	Not applicable
Private (fee-paying)	1837 (33.0)	860 (32.6)		
Other/missing	379 (6.8)	92 (3.5)		
Lifetime mental health, *n* (%)				
No diagnosis in lifetime	3074 (55.3)	1739 (66.0)	Not applicable	Not applicable
Previous mental health diagnosis	1562 (28.1)	884 (33.5)		
Missing	926 (16.7)	14 (0.5)		
Disability, *n* (%)				
Physical disability	106 (1.9)	57 (2.2)	3049 (12.2)^b^	3411 (13.1)
Non-physical disability	1283 (23.1)	581 (22.0)		
Physical and non-physical	68 (1.2)	62 (2.4)		
None	3819 (68.7)	1724 (65.4)	21 846 (87.7)	22 624 (86.8)
Prefer not to say/missing	286 (5.2)	213 (8.1)	20 (0.1)	18 (0.1)
Year of study, *n* (%)^c^				
1	1817 (32.7)	952 (36.2)	10 695 (42.9)	11 394 (43.7)
2	1605 (28.9)	692 (26.3)	6314 (25.3)	6433 (24.6)
3	1402(25.2)	583 (22.2)	5594 (22.5)	5833 (22.3)
4 and above	639 (11.5)	353 (13.4)	2243 (9.0)	2307 (8.8)
Other/missing	99 (1.8)	57 (2.2)	76 (0.3)	104 (0.4)
Faculty of study, *n* (%)				
Arts	1238 (22.3)	544 (20.6)	4851 (19.5)	4952 (19.0)
Engineering	661 (11.9)	273 (10.4)	3430 (13.8)	3504 (13.4)
Health sciences	760 (13.7)	442 (16.7)	3275 (13.1)	3368 (12.9)
Life sciences^d^	448 (8.1)	364 (13.8)	2800 (11.2)	2994 (11.5)
Science	1271 (22.9)	446 (16.9)	3543 (14.2)	3578 (13.7)
Social science and law	1141 (20.5)	557 (22.1)	7017 (28.1)	7662 (29.4)
Missing	43 (0.8)	11 (0.4)	Not applicable	Not applicable
Residence, *n* (%)				
Hall of residence	1728 (31.1)	861 (32.7)	Not applicable	Not applicable
Private rental	3496 (62.9)	1511 (57.3)		
Other/missing	338 (6.1)	265 (10.1)		

a. Institution data are reported as home/European Union combined.

b. Institution data include any disclosed disability at registration.

c. Year of study may include postgraduate or undergraduate.

d. Known as biomedical science in 2018.

#### Depression, anxiety and well-being

Mental health outcomes were similar in 2018 and 2019 for both percentage categorised and mean values (Table 2). The proportion of respondents reporting moderate/severe depression symptoms increased between 2018 (45.0%) and 2019 (46.9%); however, the corresponding proportion of students reporting moderate/severe levels of anxiety decreased (38.6% in 2018; 36.3% in 2019). There was also a decrease in students reporting low levels of mental well-being between years (51.0% in 2018; 48.5% in 2019).

**Table 2:** Dichotomous and continuous mental health outcomes in 2018 and 2019

Survey year	2018	2019
Depression (*n* = analytic sample/eligible respondents), *n* (%)	4869/5570	2602/2637
PHQ-9 <10 (no/mild symptoms)	2679 (55.0)	1383 (53.2)
PHQ-9 ≥10 (moderate/severe symptoms)	2190 (45.0)	1219 (46.9)
Anxiety (*n* = analytic sample size), *n* (%)	4696/5570	2610/2637
GAD-7 <10 (no/mild symptoms)	2885 (61.4)	1663 (63.7)
GAD-7 ≥10 (moderate/severe symptoms)	1811 (38.6)	947 (36.3)
Mental well-being (*n* = analytic sample size), *n* (%)	5115/5570	2597/2637
WEMWBS >42 (moderate/high mental well-being)	2509 (49.1)	1337 (51.5)
WEMWBS ≤42 (low mental well-being)	2606 (51.0)	1260 (48.5)
Average mental health scores, mean (s.d.)		
Depression (PHQ-9)	9.59 (6.14)	9.88 (6.70)
Anxiety (GAD-7)	8.33 (5.54)	8.04 (5.85)
Mental well-being (WEMWBS)	42.4 (9.95)	43.0 (10.37)

Mean scores are reported for cross-sector comparison only. PHQ-9, Patient Health Questionnaire-9; GAD-7, Generalised Anxiety Disorder-7; WEMWBS, Warwick–Edinburgh Mental Wellbeing Scale.

The adjusted models in Table 3 show no statistical evidence for any change in symptoms of depression (odds ratio 1.05, 95% CI 0.93–1.17) between 2018 and 2019. However, there was a 14% drop in the odds of students experiencing greater anxiety symptoms (odds ratio 0.86, 95% CI 0.77–0.96), and a 16% drop in odds of respondents experiencing low mental well-being (odds ratio 0.84, 95% CI 0.75–0.94).

**Table 3:** Adjusted and unadjusted logistic regression examining change in mental health outcomes 2018 and 2019

	Depression symptoms (PHQ-9 ≥10)		Anxiety symptoms (GAD-7 ≥10)		Poor mental well-being (WEMWBS ≤42)	
	Unadjusted *n* = 7471	Adjusted^1^ *n* = 6699	Unadjusted *n* = 7306	Adjusted^1^ *n* = 6709	Unadjusted *n* = 7712	Adjusted^1^ *n* = 6693
Survey year	Odds ratio (95% CI)	Odds ratio (95% CI)	Odds ratio (95% CI)	Odds ratio (95% CI)	Odds ratio (95% CI)	Odds ratio (95% CI)
2018 (Reference)	1.00	1.00	1.00	1.00	1.00	1.00
2019	1.08 (0.98–1.19)	1.05 (0.93–1.17)	0.91 (0.82–1.00)	0.86 (0.77–0.96)	0.91 (0.83–1.00)	0.84 (0.75–0.94)
*P*-value	0.122	0.434	0.054	0.009**	0.044*	0.002**

PHQ-9, Patient Health Questionnaire-9; GAD-7, Generalised Anxiety Disorder-7; WEMWBS, Warwick–Edinburgh Mental Wellbeing Scale.

*P-value: <0.05, **P-value: <0.01.

1 Models adjusted for gender, age, ethnicity, fee status, sexual orientation, previous education, faculty of study, year of study, lifetime mental health diagnosis, disability, residence and course level.

For comparison, the unadjusted models offered no evidence for change in odds of greater depression symptoms (odds ratio 1.08, 95% CI 0.98–1.19) or anxiety (odds ratio 0.91, 95% CI 0.82–1.00) between 2018 and 2019, and weaker evidence for a 9% drop in odds of students having low mental well-being (odds ratio 0.91, 95% CI 0.83–1.00).

#### Mental health outcomes stratified by students’ sociodemographic, health and educational characteristics

Interaction test *P*-values indicated differential effects between 2018 and 2019 in depression symptoms related to sexual orientation (*P* = 0.013) and student mental well-being associated with gender (*P* = 0.011) (see Supplementary File 4). Stratified analyses offered some evidence for LGB(TQ) students showing increased odds of greater depression symptoms between 2018 (odds ratio 1.34, 95% CI 1.12–1.61) and 2019 (odds ratio 2.08, 95% CI 1.63–2.63), compared with students identifying as heterosexual. There was also some evidence for improved mental well-being in respondents identifying as non-binary or another gender (compared with male gender as the reference group) between 2018 (odds ratio 3.46, 95% CI 1.49–8.07) and 2019 (odds ratio 0.64, 95% CI 0.27–1.51). Students who did not disclose their gender showed a similar pattern.

#### Help-seeking behaviour

The proportion of students seeking university support in their first year of study increased in 2019, with the exception of students using an online support community (Togetherall) and the Students’ Union (Supplementary File 5). The most widely used sources of support were mental health professionals (20.5% in 2018; 23.6% in 2019), general practitioners/doctors (18.8% in 2018; 28.2% in 2019) and personal tutors/academic mentors (17.7% in 2018; 25.3% in 2019). The proportion of students seeking support from staff in residences increased between 2018 (6.7%) and 2019 (10.8%). In 2019, 13.1% of students reported seeking help from the new well-being advisers in academic departments.

#### Barriers to seeking support

The most frequently reported support-seeking barrier for students in their first year of study was ‘fear of unwanted intervention’ in both 2018 (18.1%) and 2019 (23.3%) (see Supplementary File 6). The most frequently reported support-seeking barrier in 2018 for all students (18.5%) and students showing severe major depression (PHQ-9 screening score >20) (35.4%) was ‘lack of available services’. In 2019, that was replaced by ‘fear of unwanted intervention’ for all students (20.1%) and ‘concern no-one would understand the problem’ for students showing severe major depression (35.7%).

Table 4 shows an improvement in perception of structural barriers to seeking university support for students in their first year of study between 2018 and 2019. They showed lower odds of citing a ‘lack of available services’ (odds ratio 0.40, 95% CI 0.30–0.53) or ‘difficulty with access to care’ (odds ratio 0.59, 95% CI 0.44–0.77), but no corresponding change in ‘did not know where to find help’ (odds ratio 1.07, 95% CI 0.85–1.34). Assessment of all students’ experience showed improved perception of service availability (odds ratio 0.59, 95% CI 0.51–0.67), service accessibility (odds ratio 0.67, 95% CI 0.57–0.78) and not knowing where to find help (odds ratio 0.85, 95% CI 0.74–0.98). Students showing severe major depression reported improved odds in perception of service availability in 2019 (odds ratio 0.47, 95% CI 0.32–0.70), but not for accessibility (odds ratio 1.03, 95% CI 0.69–1.54). This group showed increased risk of not knowing where to find help (odds ratio 1.60, 95% CI 1.07–2.39) and had greater concerns about confidentiality (odds ratio 1.83, 95% CI 1.13–2.96).

**Table 4:** Adjusted and unadjusted models of change in perceived help-seeking barriers between 2018 and 2019

	Students in first year of study, *n* = 2769/8199 (33.8%)		All students, *n* = 8199/50 968 (16.0%)		Students showing severe major depression (PHQ-9 ≥ 20), *n* = 695/8199 (8.4%)	
Barriers to seeking help	Unadjusted odds ratio (95% CI), *P-*value	Adjusted^1^ odds ratio (95% CI), *P-*value	Unadjusted odds ratio (95% CI), *P-*value	Adjusted odds ratio (95% CI), *P-*value	Unadjusted odds ratio (95% CI), *P-*value	Adjusted odds ratio (95% CI), *P-*value
Lack of time	1.15 (0.93–1.41)	0.87 (0.69–1.09)	1.21 (1.07–1.36)	0.92 (0.81–1.05)	1.41 (1.00–1.99)	1.4 (0.99–2.13)
	0.193	0.212	0.002**	0.232	0.047*	0.054
Lack of confidentiality	1.52 (1.16–1.99)	1.22 (0.90–1.67)	1.34 (1.13–1.60)	0.99 (0.82–1.20)	1.83 (1.20–2.80)	1.83 (1.13–2.96)
	0.002**	0.289	0.001**	0.934	0.005**	0.014*
Concern ‘no-one will understand my problem’	1.33 (1.09–1.63)	1.11 (0.89–1.39)	1.23 (1.09–1.40)	0.98 (0.85–1.12)	1.35 (0.98–1.87)	1.31(0.91–1.89)
	0.006**	0.364	0.001**	0.732	0.068	0.143
Did not know where to find help	1.26 (1.03–1.56)	1.07 (0.85–1.34)	1.03 (0.91–1.18)	0.85 (0.74–0.98)	1.48 (1.03–2.11)	1.60 (1.07–2.39)
	0.027*	0.564	<0.001***	0.023*	0.032*	0.022*
Stigma of mental health care	1.06 (0.85–1.32)	1.01 (0.79–1.28)	1.14 (1.00–1.30)	0.95 (0.82–1.09)	1.12 (0.80–1.57)	0.97 (0.66–1.43)
	0.618	0.964	0.050*	0.439	0.510	0.918
Fear of unwanted intervention	1.37 (1.14–1.67)	1.18 (0.96–1.46)	1.26 (1.12–1.42)	1.03 (0.91–1.18)	1.26 (0.91–1.74)	1.20 (0.83–1.74)
	0.001**	0.156	<0.001***	0.584	0.167	0.329
Fear of documentation	1.47 (1.19–1.81)	1.25 (0.99–1.57)	1.27 (1.12–1.44)	0.98 (0.86–1.13)	1.11 (0.80–1.54)	1.06 (0.73–1.55)
	<0.001***	0.060	<0.001***	0.817	0.538	0.756
Difficulty with access to care	0.72 (0.56–0.92)	0.59 (0.44–0.77)	0.87 (0.76–1.00)	0.67 (0.58–0.78)	1.04 (0.73–1.48)	1.03 (0.69–1.54)
	0.010*	<0.001***	0.053	<0.001***	0.831	0.880
Lack of available services	0.54 (0.43–0.69)	0.40 (0.30–0.53)	0.76 (0.67–0.87)	0.59 (0.51–0.67)	0.55 (0.39–0.78)	0.49 (0.33–0.73)
	<0.001***	<0.001***	<0.001***	<0.001***	0.001**	<0.001***
Other	0.83 (0.65–1.07)	0.69 (0.53–0.91)	0.77 (0.65–0.86)	0.60 (0.52–0.70)	0.63 (0.41–0.98)	0.58 (0.36–0.94)
	0.146	0.009*	<0.001***	<0.001***	0.042*	0.027*

*P-value: <0.05, **P-value: <0.01, ***P-value: <0.001.

1 Models adjusted for gender, age, ethnicity, fee status, sexual orientation, previous education, faculty of study, year of study, lifetime mental health diagnosis, disability, residence and course level.

#### Administrative findings

##### Antidepressant prescribing

**Fig. 2:**
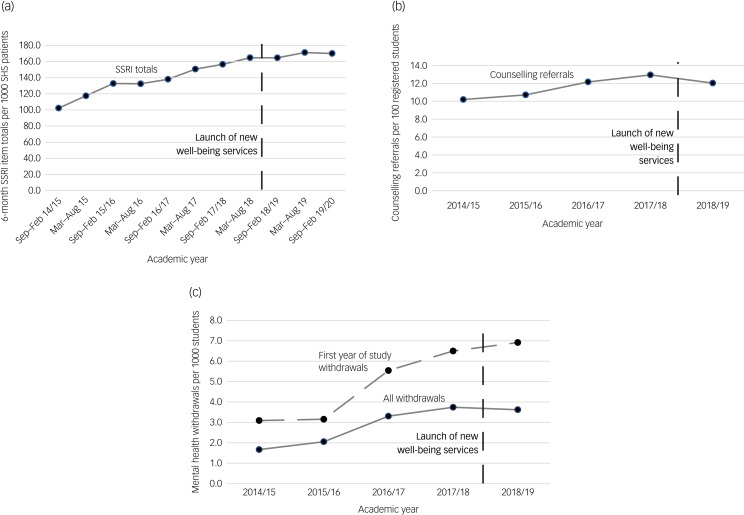
(a) Antidepressant prescribing totals, (b) annual student counselling service referral totals and (c) annual course withdrawals for mental health reasons. SHS, student health services; SSRI, selective serotonin reuptake inhibitor.

There was a 66.2% increase in (6-month) total SSRI items prescribed (per 1000 practice-registered students) between September 2014 and February 2020 (see Fig. 2(a)). However, inspection of Fig. 2(a) suggests a levelling off in 2018–2019 after the new support introduction. The average annual prescribing increase between 2014–2015 and 2018–2019 was 11.4%, but fell to its lowest level (4.5%) between 2017–2018 and 2018–2019 (Supplementary File 7).

##### Student counselling referrals

Figure 2(b) shows a levelling off in referral numbers after the introduction of the new services in September 2018 (see also Supplementary File 8). There was statistical evidence for decreased student referral numbers between 2017–2018 and 2018–2019 (risk ratio 0.93, 95% CI 0.88–0.97; *P =* 0.004).

##### Student course withdrawals

There was a steady increase in numbers of students dropping out of their courses for mental health reasons between 2015–2016 and 2017–2018, which appeared to level off for all students after the introduction of the new services in 2018–2019 (Fig. 2(c)). However, there was no statistical evidence of a difference in mental health-related course withdrawals between 2017–2018 and 2018–2019, either for all students (risk ratio 0.97, 95% CI 0.72–1.30; *P =* 0*.82*) or students in their first year of study (risk ratio 1.06, 95% CI 0.75–1.51;, *P =* 0*.72*).

## Discussion

This is, to our knowledge, the first study to examine changes in overall student mental health and help-seeking behaviour after significant well-being investment at a higher education institution. Following the introduction of new non-clinical well-being advisers at a UK university in 2018–2019, our findings point to a reduction in overall levels of student anxiety and low mental well-being, but no corresponding change in depression symptoms. At the same time, a shift in student perception of barriers to seeking help suggests university support was seen overall as more accessible and available in 2019.

Taken together, these findings point to the service's introduction having a number of positive direct and indirect effects. Namely, the reduction in overall levels of anxiety and low well-being (but not depression) may be a consequence of students having received timely well-being support when they actually sought it, or simply feeling reassured that university support was more readily accessible if they needed it. Recent studies exploring priorities for university mental health services have underlined service accessibility and availability as key issues for students, and the addition of new well-being support at this institution may have met those concerns.^32,33^ Theories of behaviour change would support the premise that highly visible new well-being investment and service promotion might serve as a vehicle for ‘nudging’ or positively influencing the broader student support experience, leading to improved student confidence in university support, regardless of need.^34,35^ Indeed, a marked positive shift in student and staff perception of the university's support provision was also seen in qualitative findings from focus groups and interviews carried out as part of the wider evaluation.^25^ It strengthens the case for the new intervention having had a positive impact on student levels of anxiety and low well-being overall.

In the absence of comparator university data, findings from administrative data offer further support for positive secondary effects of the new investment – most clearly evidenced by a decrease in student counselling referrals in 2019. This reduction suggests the new services were addressing a ‘lack of available services’ or ‘long wait times’ for university support.^13,17,36^ Fewer counselling referrals would also have met the institution's strategic aim for the new advisers to free up high-intensity clinical provision, easing welfare pathways and pressure on other university staff.^24^ That said, the increased numbers of students seeking help from mental health professionals more broadly, as well as from general practitioners and tutors/mentors, between 2018 and 2019, suggests that the new services might not have reduced pressure on academics or wider health services.

A further positive indication of downstream effects of the new advisers was seen in a descriptive levelling off of antidepressant prescribing and student course withdrawals for mental health reasons between 2018 and 2019, although this evidence is notably weaker. Finally, although there was some evidence for differential effects of the new support introduction, the number of analyses make these results less convincing. Any reasons for improved well-being for minority gender students in 2019 or worsening in depression symptoms in LGBTQ students as a result of the new service provision are also unclear. The absence of clear evidence suggests no particular student group was advantaged or disadvantaged by the introduction of the new services.

### Strengths and limitations

This was a pragmatic study, examining impact for a whole university population, not simply those using services. Strengths are its novelty, availability of pre-intervention survey data and breadth of scope: critically, it is some of the first research evidence in higher education to report on the impact of large-scale well-being support investment. However, there are notable limitations. Cross-sectional surveys with low response rates may be affected by non-response bias, i.e. the characteristics of those responding may differ from the wider student population and between higher and lower response rate surveys.^37^ The survey was administered by the Students’ Union in 2018 and the university in 2019, thus the notable drop in response rates may have been affected by differences in recruitment periods and differing advertising resources. In our analyses, we controlled for differences in responder characteristics to mitigate the impact of differing samples; however, there is still potential for further differences between the waves of data collection to have influenced our results in ways not considered here.

Additionally, without comparison universities or longer time-trends analyses (rendered impossible by COVID-19-related disruption), our findings may reflect transitory features such as background changes in mental health, SSRI prescribing trends or changes in course withdrawal reporting, rather than robust intervention effects. Likewise, it may reflect a ‘honeymoon period’, after which new services can be overwhelmed by demand, something suggested by more recent evidence from the service providers. In the second year of operation, with the further introduction of a triaging system into the new services, more than 6000 students were seen in the first 4 months alone, compared with the 5000 seen in the whole first year.^24^ Although there are no comparable UK longitudinal data, both government reporting and similar-age cohort studies do suggest that young people's mental health outcomes were deteriorating over the same period as this study.^38–40^ On the other hand, one large USA student cohort study spanning 2013 to 2021 that used the same mental health measures, suggests that the only academic year in which student mental health outcomes improved or stayed the same was 2018–2019.^2^

A further limitation is the size of some of our models and, specifically, our examination of the varying effects of the new service introduction on different student groups. The large number of analyses (*n* = 27) may have increased the risk of type 1 error, with small sample sizes resulting in wide confidence intervals. A final issue is comparability: this was a single-site study in an education sector with many differing support frameworks and geo-political contexts, making generalising challenging.^14,41^

### Future directions

Higher education is a critical environment in which universally applied population health interventions can have a significant impact on young people's mental health and well-being, and it is vital that future research focuses on interventions that might have the broadest reach.^7,42,43^ Systematic mental health data collection in this population is critical – ideally longitudinal, linked datasets, with some degree of standardisation across mental health measures.^37,44^ Despite significant investment in student mental health research by organisations such as the Office for Students and UK Research and Innovation (UKRI), to date there is still no current commitment to national longitudinal data collection.^45,46^

Careful evaluation needs to be built into the future design and development of all new or restructured well-being support in higher education, with findings more widely shared and disseminated across the sector. It is likely that many higher education providers already carry out internal audits and process evaluations without sharing good practice beyond their leadership teams or institutions. Population health evaluation across broad contexts will be critical if universities are serious about creating healthy environments for students and staff, as opposed to stemming the tide. That includes working with schools in secondary education settings, the National Health Service and private student accommodation providers.

In conclusion, this study addresses a critical gap in the existing literature and offers preliminary evidence for the impact of non-clinical well-being advisory teams in higher education. Although it is a single university study, there is broader learning for the wider sector regarding how a low-intensity, stepped-care model of student support can transform student perception of the availability and accessibility of services, reduce need for clinical intervention and potentially influence overall levels of student anxiety and well-being.

## Supporting information

Bennett et al. supplementary material Bennett et al. supplementary material

## Data Availability

The data that support the findings of this study are available from the corresponding author, J.B., upon reasonable request.
