# Supplementary material for: Investigating changes in student mental health and help-seeking behaviour after the introduction of new well-being support services at a UK university
Source: BJPsych Open. 2024 May 27;10(3):e121. doi: 10.1192/bjo.2024.711 (PMC11363082; doi:10.1192/bjo.2024.711)
Supplement: Bennett et al. supplementary material [file S2056472424007117sup001.docx]

**Supplementary Files**

1. Wellbeing and Residence Life Adviser Recruitment and Training

2. Survey Measures and Student Characteristics (Full questionnaire available from author on request)

3. Un/Adjusted Regression Models Examining Missing Mental Health Diagnosis Survey Items

4. Interaction P-values for Differential Effects of New Wellbeing Services on Mental Health Outcomes

5. Use of University Support in 2018 and 2019 by Students in First Year of Study

6. Experience of Barriers to Seeking University Support in Students in First Year of Study, All Respondents and Students showing Severe Major Depression (SMD)

7. Annual % Change in SSRI Items Prescribed at Student Health Service 2014 to 2019

8. Numbers of Student Counselling Service Referrals and Registered Students 2014 to 2019

1. Wellbeing and Residence Life Adviser Recruitment and Training

*Excerpt from Job Specification in 2018.*

“2.2 Relevant Qualifications

Essential

• Vocational qualifications (NVQ4, HNC or HND in a relevant subject), or equivalent qualifications plus considerable experience in a relevant role(s)

Desirable

• Relevant professional training e.g. coaching, social work, counselling, teaching etc”

*Training Courses for newly recruited Wellbeing and Residence Life Advisers included:**

External Training:

Motivational Interviewing

Brief Solution Focused Therapy

Mental Health First Aid

ASIST

Bereavement training

Eating Disorders Training

Internal Training:

Basic Counselling Skills

Introductory sessions from Disability, Careers, Residence Life, Student Health Service, Mental Health Advisory Service, Student Counselling, Chaplaincy, Inclusion Team, Student Recruitment, Students’ Union.

**List included as a general guide and is not exhaustive*

1. Survey Measures and Student Characteristics (Full questionnaire available from author on request)

| **Outcome** | **Measure** | | **Sample item** | | **Scoring** | **Score Meaning** | |
| --- | --- | --- | --- | --- | --- | --- | --- |
| ***Mental health*** |  | |  | |  |  | |
| **Depression - screens for symptoms** | PHQ-9  Patient Health Questionnaire^1^ | | In the last two weeks how often have you been bothered by any of the following:  *Little interest or pleasure in doing things?*  *Feeling down, depressed, or hopeless? etc* | | Not at all (0) Several Days (1) More than half the days (2)  Nearly every day (3) | Depression symptoms:  1-4 None  5-9 Mild  10-14 Moderate  15-19 Moderately Severe  20-27 Severe  Score ≥ 10 merits further clinical investigation in a primary care setting | |
| **Anxiety -screens for symptoms** | GAD-7  Generalised Anxiety Scale^2^ | | In the last two weeks how often have you been bothered by any of the following:  *Feeling nervous anxious or on edge?*  *Not being able to stop or control worrying? etc* | | As above | As above  Scores are between 1-21 and score ≥ 10 merits further clinical investigation | |
| **Mental Well-being** | WEMWBS  Warwick and Edinburgh Mental Wellbeing Scale^3^ | | Please tick the box that best describes your experience in the last two weeks:  *I’ve been feeling optimistic about the future*  *I’ve been feeling useful*  *I’ve been feeling relaxed*  *I’ve been feeling interested in other people etc* | | None of the time (1)  Rarely (2)  Some of the time (3)  Often (4)  All of the time (5) | Scores between 14-70 with higher score indicating more positive well-being and recommended cut off < 42 indicating low wellbeing-equivalent to the lowest 15% of scores in the general population. Average UK general population score is 51.0, SD 7. A meaningful difference considered between 3 and 8.^4^ | |
| ***Help-seeking behaviour*** |  | |  | |  |  | |
| **Support networks used** | List of 11 university support options | | a) Which of the following have you **ever** sought help from since starting university?  Options included: *Staff in Residences, Well-being Adviser, Mental Health Professional, University Support Staff, GP/Doctor, Peer Support, Students’ Union Adviser, Personal Tutor/Academic mentor, Other Academic Staff, Togetherall (formerly Big White Wall - an online support community), Nightline (phone support)* | | a) Yes, No, Not applicable. | Frequencies and percentages | |
| **Perceived barriers to help-seeking** | List of barriers^5,6^ | | If you have had a mental health or wellbeing concern and have not used the university’s support services, please indicate why:  **Not had a problem*  **Lack of time*  **Lack of confidentiality*  **Concern that "no one will understand my problems"*  **I didn't know where to find help*  **Stigma of mental health care*  **Fear of unwanted intervention*  **Fear of documentation on academic record*  **Difficulty with access to care*  **Lack of available services*  **Other* | | (Could tick all that applied) | Frequencies and percentages | |
| **Confounder/Interaction effect modifier** | | **Measure** | | **Inclusion rationale** | | | **Coding** |
| ***Student characteristic*** | |  | |  | | |  |
| **Gender** | | Gender identity using the Office for National Statistics categorisations^7^ | | Females and minority genders at greater risk of mental health (MH) issues^8,9^ | | | Man (reference), Woman, Non-binary/Another gender, Prefer not to say (PNS) |
| **Age** | | Age in years | | Students over 21 years are considered mature students with known age-related HE barriers and potential differential effects of intervention and service use ^10,11^ | | | **≥** 21 years (reference) and < 21 |
| **Ethnicity** | | Ethnicity using Office for National Statistics categories ^12^ ^a^ | | Minority ethnicity background associated with poorer outcomes^13^ | | | White ethnicity (reference), Minority ethnicity, and PNS |
| **Sexual Orientation** | | Sexual identity, attraction or behaviour using Office for National Statistics categories^14^ | | Minority sexual orientation associated with poorer MH outcomes^15^ | | | Heterosexual/Straight (reference), Lesbian/Gay/Bisexual (LGB), PNS |
| **International or Home** | | Fee status | | Cultural challenges for overseas students^16,17^ | | | Home/ Channel Isles (reference) and EU/International |
| **Level of study** | | Course level | | PGR associated with better MH outcomes, PGT and UG poorer MH^18^ | | | Undergraduate (UG) (reference), Postgraduate Taught (PGT), Postgraduate Researcher (PGR) |
| **Social Economic Indicator** | | Previous Education/Schooling | | Disadvantaged background as a predictor for MH concerns^19^ | | | State - non-feepaying (reference), Grammar - non-fee paying and Other, Private or grammar - fee-paying |
| **Previous (Lifetime) Mental health diagnosis** | | Previous or current mental health concerns - sometimes called ‘lifetime mental health (MH) diagnosis’ | | Lifetime MH diagnosis associated with CMD 12 month prevalence and suicidality^20,21^ | | | No (reference)  Yes |
| **Disability** | | Physical and non-physical disability in line with institution’s classifications | | Associated with MH concerns^22^ | | | None (reference), Physical, Non-physical, Both,  PNS |
| **Year of study** | |  | | Transition/first year of study and association with MH concerns^23^ | | | Year 1 (reference), Year 2, Year 3, Year 4, Year 5/6, Other |
| **Faculty of study** | | Overarching educational discipline or subject area comprising a number of separate schools (or sub-divisions) e.g., Faculty of Arts contains School of English | | Arts and Social Science studies association with poorer MH outcomes^24^ | | | Arts (reference), Life Sciences, Engineering, Health Sciences, Science, Social Science and Law |
| **Place of residence** | |  | | Hall of residence as a predictor for MH concerns^25^ | | | University run hall (reference), Private Hall, Private landlord, Other |

*^a^ Minority ethnicity backgrounds grouped together to maximise limited sample*

|  |  |  |  |  |  |  |  |
| --- | --- | --- | --- | --- | --- | --- | --- |

1. Un/Adjusted Regression Models Examining Missing Mental Health Diagnosis Survey Items

| **Sample size n=6,648/8,199** | **OR (CI 95%)** | ***p value*** |
| --- | --- | --- |
| **Depression symptoms**  (PHQ9 ≥10) | | |
| 2018 (ref) | 1.00 | *1.00* |
| 2019 Unadjusted ^1^ | 1.08 (0.98-1.19) | *.122* |
| 2019 Adjusted Model ^2^ | 1.03 (0.92-1.15) | *.617* |
| 2019 Adjusted Model ^3^ | 1.05 (0.93-1.17) | *.434* |
| **Anxiety Symptoms**  (GAD7 ≥ 10) | | |
| 2018 (ref) | 1.00 | *1.00* |
| Unadjusted ^1^ | 0.91 (0.82-1.00) | *.054* |
| 2019 Adjusted Model ^2^ | 0.85 (0.76-0.95) | *.006** |
| 2019 Adjusted Model ^3^ | 0.86 (0.77-0.96) | *.010** |
| **Mental Wellbeing**  (WEMWBS ≤42) | | |
| 2018 (ref) | 1.00 | *1.00* |
| Unadjusted ^1^ | 0.91 (0.83-1.00) | *.044** |
| 2019 Adjusted Model ^2^ | 0.83 (0.75- 0.93) | *.001** |
| 2019 Adjusted Model ^3^ | 0.84 (0.75-0.94) | *.002*** |

1 Model with no confounders

2 Model adjusted for all confounders omitting lifetime MH diagnosis

3 Model adjusted for all confounders including lifetime MH diagnosis

1. *Interaction P-values for Differential Effects of New Wellbeing Services on Mental Health Outcomes*

| **Risk factor** ^1^ | **Depression Symptoms**  **(PHQ>10)** | **Anxiety Symptoms**  **(GAD>10)** | **Mental Wellbeing**  **(WEMWBS <42)** |
| --- | --- | --- | --- |
|  | *Interaction p value* | *Interaction p value* | *Interaction p value* |
| **Lifetime MH diagnosis** | .498 | .412 | .557 |
| **State Educated** | .868 | .274 | .958 |
| **International** | .223 | .208 | .366 |
| **Gender** | .465 | .612 | .011* |
| **Ethnicity** | .444 | .662 | .592 |
| **Sexual orientation** | .013* | .866 | .808 |
| **Year of study** | .394 | .781 | .354 |
| **Course Level** | .146 | .404 | .663 |
| **Faculty of study** | .702 | .548 | .670 |
| **Disability** | .193 | .250 | .142 |

1 all models adjusted for: *gender, ethnicity, fee status, sexual orientation, previous education, faculty of study, year of study, lifetime MH diagnosis, disability, course level*

1. *Use of University Support in 2018 and 2019 by Students in First Year of Study*

a Axis censored at 50% to accommodate smaller values

b All staff in residences collapsed into one category

c No Wellbeing advisers in 2018

1. *Experience of Barriers to Seeking University Support in Students in First Year of Study, All Respondents, and Students showing Severe Major Depression (SMD)*

** Y-axis censored at 50% to accommodate smaller values*

1. *Annual % Change in SSRI Items Prescribed at Student Health Service 2014 to 2019*

| **Academic years** | **% Annual SRRI total change (per 1,000 SHS registered students)** |
| --- | --- |
| 2014/15 to 2015/16 | +20.7% |
| 2015/16 to 2016/17 | +8.8% |
| 2016/17 to 2017/18 | +11.3% |
| 2017/18 to 2018/19 | +4.5% |

1. *Numbers of Student Counselling Service Referrals and Registered Students 2014 to 2019*

| **Academic year** | **Counselling referrals**  n= total students (% population) | **Student population**  n=total |
| --- | --- | --- |
| **2014/15** | 2192 (10.2) | 21,500 |
| **2015/16** | 2350 (10.7) | 21,945 |
| **2016/17** | 2889 (12.2) | 23,764 |
| **2017/18** | 3225 (12.9) | 24,915 |
| **2018/19** | 3134 (12.0) | 26,053 |

**Supplementary references**

1. Kroenke K, Spitzer RL, Williams JB. The PHQ‐9: validity of a brief depression severity measure. J Gen Intern Med. 2001;16(9):606-613. [DOI: 10.1046/j.1525-1497.2001.016009606.x]
2. Spitzer RL, Kroenke K, Williams JB, Löwe B. A brief measure for assessing generalized anxiety disorder: the GAD-7. Arch Intern Med. 2006;166(10):1092-1097.
3. Tennant R, Hiller L, Fishwick R, Platt S, Joseph S, Weich S, Parkinson J, Secker J, Stewart-Brown S. The Warwick-Edinburgh mental well-being scale (WEMWBS): development and UK validation. Health Qual Life Outcomes. 2007;5(1):1-13. doi: 10.1186/1477-7525-5-63
4. Warwick Medical School. Collect, score, analyse and interpret WEMWBS. [Internet]. 2021 <https://warwick.ac.uk/fac/sci/med/research/platform/wemwbs/using/howto/>
5. Knipe D, Maughan C, Gilbert J, Dymock D, Moran P, Gunnell D. Mental health in medical, dentistry and veterinary students: cross-sectional online survey. BJPsych Open. 2018;4(6):441-446. [DOI: 10.1192/bjo.2018.61]
6. Thorley C. Not by Degrees: Improving Student Mental Health in the UK’s Universities. Institute for Public Policy Research [IPPR]. 2017. Available from: [www](http://www/). ippr.org/publications/not-by-degrees.
7. ONS. What’s the difference between sex and gender? Office for National Statistics. 2019 <https://www.ons.gov.uk/economy/environmentalaccounts/articles/whatisthedifferencebetweensexandgender/2019-02-21#definitions-and-differences>
8. Lipson SK, Raifman J, Abelson S, Reisner SL. Gender minority mental health in the US: Results of a national survey on college campuses. Am J Prev Med. 2019;57(3):293-301. [DOI: 10.1016/j.amepre.2019.04.025]
9. McManus S, Gunnell D, Cooper C, Bebbington PE, Howard LM, Brugha T, et al. Prevalence of non-suicidal self-harm and service contact in England, 2000–14: repeated cross-sectional surveys of the general population. Lancet Psychiatry. 2019;6(7):573-581. [DOI: 10.1016/S2215-0366(19)30188-9]
10. HESA. Definitions: Students. Higher Education Statistics Agency. <https://www.hesa.ac.uk/support/definitions/students>
11. OfS. Mature Students. Office for Students. 2020. <https://www.officeforstudents.org.uk/advice-and-guidance/promoting-equal-opportunities/effective-practice/mature-students/>
12. ONS. Ethnicity Facts and Figures. Office for National Statistics. [URL: <https://www.ethnicity-facts-figures.service.gov.uk/>]
13. Arday J. Understanding Mental Health: What are the Issues for Black and Ethnic Minority Students at University? Soc Sci. 2018;7(10):196. [DOI: 10.3390/socsci7100196]
14. ONS. Sexual orientation, UK: 2019. Office for National Statistics. [URL: <https://www.ons.gov.uk/peoplepopulationandcommunity/culturalidentity/sexuality/bulletins/sexualidentityuk/2019#main-points>]
15. Liu CH, Stevens C, Wong SH, Yasui M, Chen JA. The prevalence and predictors of mental health diagnoses and suicide among US college students: Implications for addressing disparities in service use. Depress Anxiety. 2019;36(1):8-17. [DOI: 10.1002/da.22830]
16. Alharbi ES, Smith AP. Studying Away and Well-Being: A Comparison Study Between International and Home Students in the UK. Int Educ Stud. 2019;12(6). [DOI: 10.5539/ies.v12n6p1]
17. Brunsting NC, Zachry C, Takeuchi R. Predictors of undergraduate international student psychosocial adjustment to US universities: A systematic review from 2009-2018. Int J Intercultural Relations. 2018;66:22-33. [DOI: 10.1016/j.ijintrel.2018.06.002]
18. Wyatt T, Oswalt SB. Comparing mental health issues among undergraduate and graduate students. Am J Health Educ. 2013;44(2):96-107. doi: 10.1080/19325037.2013.764248
19. Stebleton MJ, Soria KM, Huesman Jr RL. First‐generation students' sense of belonging, mental health, and use of counseling services at public research universities. J Coll Couns. 2014;17(1):6-20. doi: 10.1002/j.2161-1882.2014.00044.x
20. Auerbach RP, Mortier P, Bruffaerts R, Alonso J, Benjet C, Cuijpers P, et al. WHO World Mental Health Surveys International College Student Project: Prevalence and distribution of mental disorders. J Abnorm Psychol. 2018;127(7):623-638. [DOI: 10.1037/abn0000362]
21. Casey SM, Varela A, Marriott JP, Coleman CM, Harlow BL. The influence of diagnosed mental health conditions and symptoms of depression and/or anxiety on suicide ideation, plan, and attempt among college students: Findings from the Healthy Minds Study, 2018–2019. J Affect Disord. 2022;298:464-471.
22. Thorley C. Not by Degrees: Improving Student Mental Health in the UK’s Universities. Institute for Public Policy Research [IPPR]. 2017. Available from: [www](http://www/). ippr.org/publications/not-by-degrees.
23. Bruffaerts R, Mortier P, Auerbach RP, Alonso J, Hermosillo De la Torre AE, Cuijpers P, et al. Lifetime and 12‐month treatment for mental disorders and suicidal thoughts and behaviors among first year college students. e1764. Int J Methods Psychiatric Res. 2019. [DOI: 10.1002/mpr.1764]
24. Lipson SK, Zhou S, Wagner III B, Beck K, Eisenberg D. Major differences: Variations in undergraduate and graduate student mental health and treatment utilization across academic disciplines. J College Student Psychotherapy. 2016;30(1):23-41. [DOI: 10.1080/87568225.2016.1105657]
25. Brett CE, Mathieson ML, Rowley AM. Determinants of wellbeing in university students: The role of residential status, stress, loneliness, resilience, and sense of coherence. Curr Psychol. 2022;1-10. [DOI: 10.1007/s12144-022-03125-8]
